# Reactive Oxygen Species Mediate the Suppression of Arterial Smooth Muscle T-type Ca^2+^ Channels by Angiotensin II

**DOI:** 10.1038/s41598-018-21899-5

**Published:** 2018-02-22

**Authors:** Ahmed M. Hashad, Maria Sancho, Suzanne E. Brett, Donald G. Welsh

**Affiliations:** 1 Deptartment of Physiology & Pharmacology, Hotchkiss Brain and Libin Cardiovascular Institutes, University of Calgary, Alberta, Canada; 2 Deptartment Physiology & Pharmacology, University of Western Ontario, London, Ontario Canada

## Abstract

Vascular T-type Ca^2+^ channels (Ca_V_3.1 and Ca_V_3.2) play a key role in arterial tone development. This study investigated whether this conductance is a regulatory target of angiotensin II (Ang II), a vasoactive peptide that circulates and which is locally produced within the arterial wall. Patch clamp electrophysiology performed on rat cerebral arterial smooth muscle cells reveals that Ang II (100 nM) inhibited T-type currents through AT_1_ receptor activation. Blocking protein kinase C failed to eliminate channel suppression, a finding consistent with unique signaling proteins enabling this response. In this regard, inhibiting NADPH oxidase (Nox) with apocynin or ML171 (Nox1 selective) abolished channel suppression highlighting a role for reactive oxygen species (ROS). In the presence of Ni^2+^ (50 µM), Ang II failed to modulate the residual T-type current, an observation consistent with this peptide targeting Ca_V_3.2. Selective channel suppression by Ang II impaired the ability of Ca_V_3.2 to alter spontaneous transient outward currents or vessel diameter. Proximity ligation assay confirmed Nox1 colocalization with Ca_V_3.2. In closing, Ang II targets Ca_V_3.2 channels via a signaling pathway involving Nox1 and the generation of ROS. This unique regulatory mechanism alters BK_Ca_ mediated feedback giving rise to a “constrictive” phenotype often observed with cerebrovascular disease.

## Introduction

Cerebral blood flow is controlled by an integrated network of resistance arteries that actively respond to mechanical and chemical stimuli^1,2^. Vasoactive stimuli, via defined receptors and transduction pathways, influences arterial tone by altering membrane potential (V_M_) and consequently the influx of extracellular Ca^2+^ through L-type Ca^2+^ channels^3–5^. While L-type channels dominate Ca^2+^ entry, two T-type channels (Ca_V_3.x) are additionally expressed in vascular smooth muscle, each impacting tone development in a distinctive manner^6,7^. Ca_V_3.2 is particularly noteworthy in triggering Ca^2+^ sparks, localized Ca^2+^ events that activate large conductance Ca^2+^ activated K^+^ (BK_Ca_) channels, generating spontaneous transient outward currents (STOCs)^8–10^. STOCs hyperpolarize smooth muscle and are a part of a key negative feedback loop that regulates arterial constriction^11^.

The importance of Ca_V_3.2 as a regulatory target is beginning to emerge with existing observations focusing on the influence of dilatory protein kinases^12,13^. The impact of vasoconstrictors, principally those linked to BK_Ca_ regulation and the generation of negative feedback, have surprisingly escaped experimental attention. Of note is angiotensin II (Ang II), a vasoactive peptide that circulates systemically at variable levels and is produced locally in the arterial wall^14–16^. The constrictor effects of Ang II are classically tied to the AT_1_ receptor and the ability of the G_q_ signaling pathway to mobilize key molecules like protein kinase C (PKC), a phosphoprotein that targets multiple downstream effectors^17,18^. This could include Ca_v_3.2 whose inhibition would impair STOC generation, suppress negative feedback and enhance arterial constriction at physiological voltages^19–21^.

This study examined whether and by what mechanism Ang II modulates T-type Ca^2+^ channels in vascular smooth muscle. Experiments progressed from isolated smooth muscle cells to intact arteries, and involved the integrative use of patch clamp electrophysiology, vessel myography, and confocal microscopy. Findings show that AT_1_ receptor activation suppressed the Ca_V_3.x current through a signaling pathway that involved NADPH oxidase (Nox) but not PKC. Subsequent work then revealed that current suppression was specifically linked to Ca_V_3.2 channels with Nox1 being the primary isoform involved. In closing, this study is the first to draw a direct link between AT1 receptors, Nox1 mediated generation of reactive oxygen species (ROS), Ca_V_3.2 suppression and the modulation of BK_Ca_ mediated feedback.

## Results

**Figure 1:**
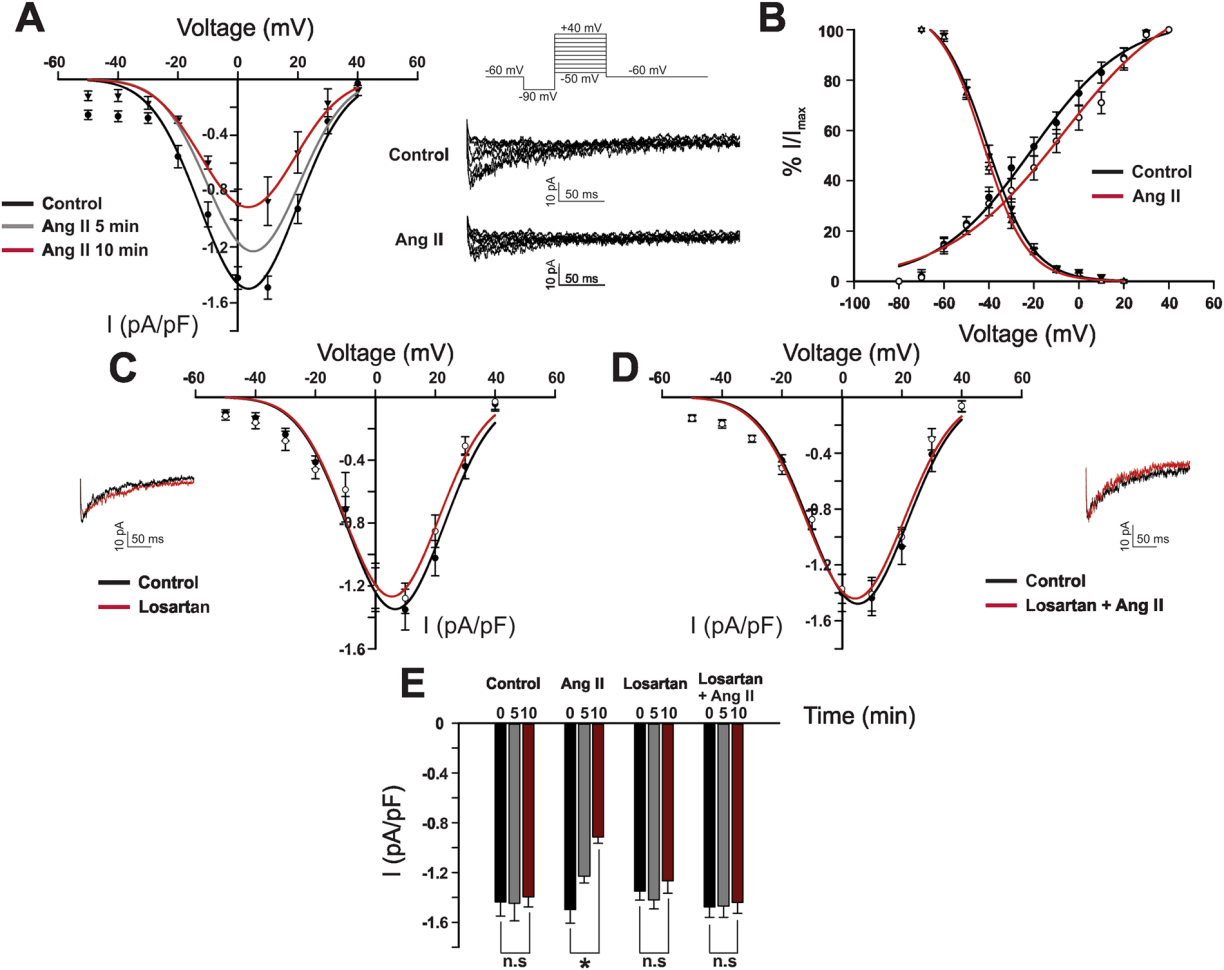
Angiotensin II inhibits T-type Ca^2+^ channels via the AT_1_ receptor in cerebral arterial smooth muscle cells. (**A** and **B**) Representative traces, averaged I-V plots, and voltage dependence of activation and steady state inactivation of whole cell T-type current prior to and following Ang II application (100 nM) (n = 8). (**C** and **D**) Representative trace and average I-V plot of the T-type current in cells treated with losartan (1 µM) and then Ang II (n = 8). (**E**) Peak T-type current (pA/pF) at 5 min intervals under control condition and following treatment with losartan ± Ang II (n = 8). All recordings were performed in the presence of 200 nM nifedipine and using Ba^2+^ as a charge carrier. *Denotes significant difference compared to control (*P < 0.05, paired t test).

Whole cell patch clamp recordings confirmed targeting of T-type Ca^2+^ channels by Ang II, a vasoconstrictive peptide present in cerebral circulation^22^. Ang II (100 nM) decreased peak inward Ba^2+^ current in a time-dependent manner (−1.4 ± 0.1 pA/pF to −1.2 ± 0.05 and 0.9 ± 0.05 pA/pF at 5 and 10 min, respectively) (Fig. 1). Additionally, Ang II drove a rightward shift trend in the voltage dependence of steady state activation (V_50_ activation, from −23.1 ± 1.7 mV to −15 ± −1.2 mV) but not steady state inactivation (V_50_ inactivation, from −42.1 ± 0.9 mV to −41 ± −0.9 mV). The ability of Ang II to suppress T-type activity was mediated via AT_1_ receptors as preincubation with 1 µM Losartan abolished all inhibitory effects (−1.47 ± 0.08 pA/pF vs −1.44 ± 0.08 pA/pF). Losartan alone had no effect on peak inward current or voltage dependence of activation/inactivation. Note, Ba^2+^ was the preferred charge carrier, as its elevated concentration promotes current flow without facilitating isolated smooth muscle cell contraction^12^.

**Figure 2:**
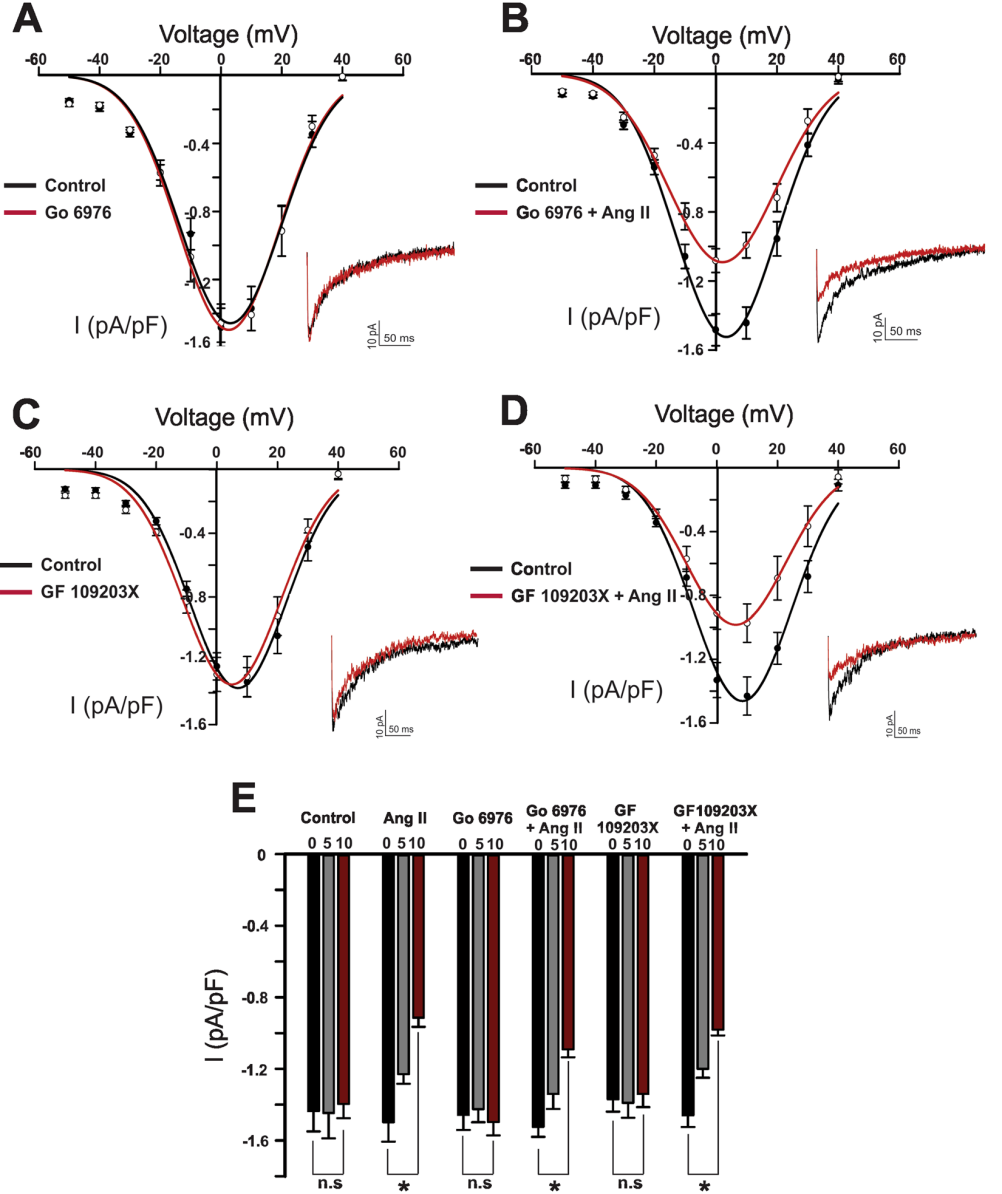
Protein Kinase C (PKC) is not involved in angiotensin II-mediated inhibition of T-type Ca^2+^ channels. (**A**) Representative trace, and averaged I-V plots of whole cell T-type current, prior to and after the application of Go 6976 (conventional PKC inhibitor, 100 nM) (n = 8). (**B**) Representative trace and average I-V plot highlighting the inability of Go 6976 to abolish Ang II (100 nM) mediated inhibition of the T-type current (n = 8). (**C**) Representative trace, averaged I-V plots of the whole cell T-type current, prior to and following the application of GF 109203X (non-selective PKC inhibitor, 100 nM) (n = 9). (**D**) Representative trace and average I-V plot highlighting the inability of GF 109203X to abolish Ang II (100 nM) mediated inhibition of the T-type current (n = 8). (**E**) Peak T-type current (pA/pF) at 5 min intervals under control condition (n = 8) and in cells treated with either Go 6976 or GF 109203X ± Ang II (100 nM) (n = 8–9). *Denotes significant difference compared to control (*P < 0.05, paired t test).

**Figure 3:**
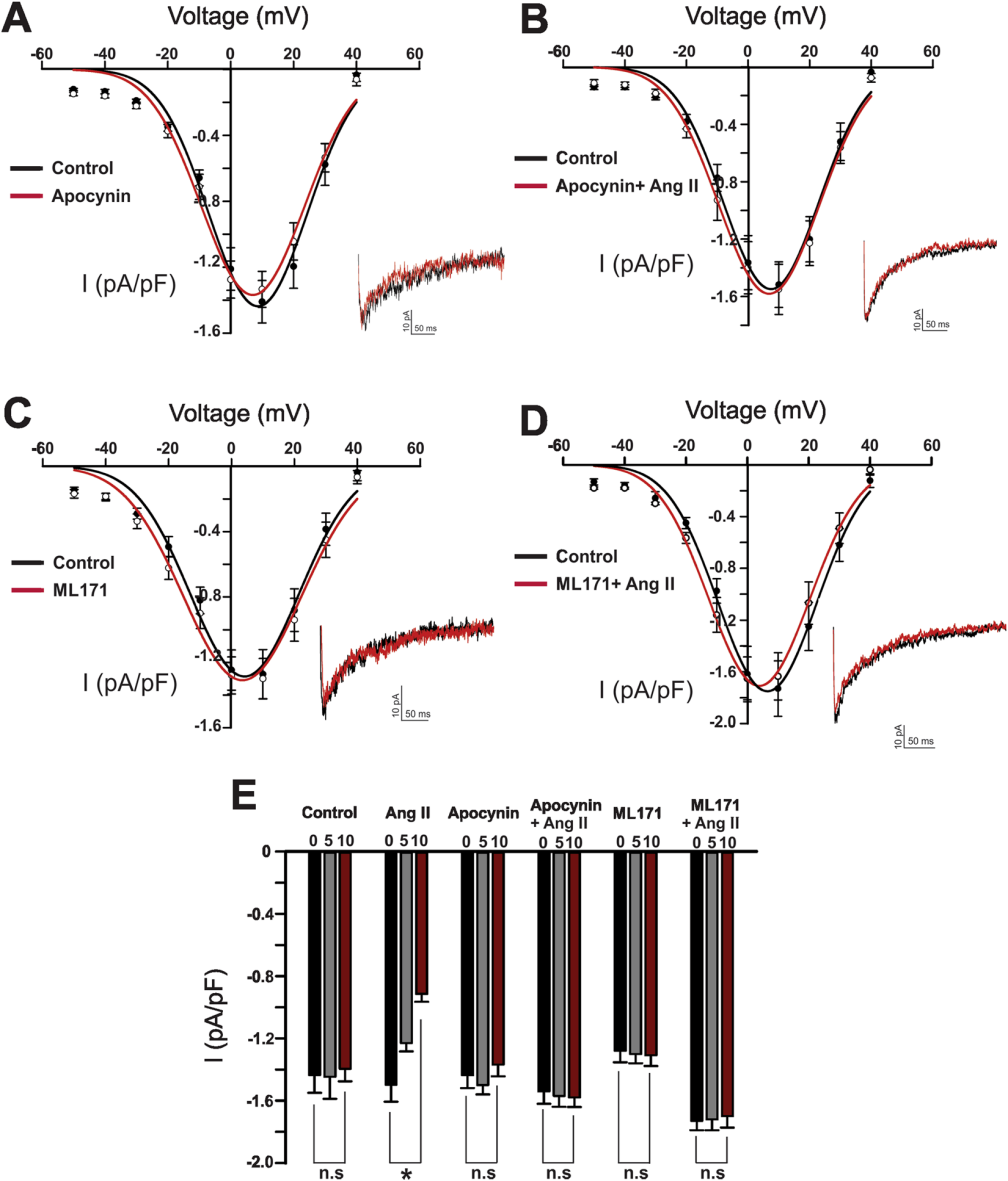
NADPH oxidase (Nox) inhibition abolishes angiotensin II suppression of the T-type Ca^2+^ current. (**A**) Representative trace, and averaged I-V plots of the whole cell T-type current, prior to and following the application of apocynin (non-selective Nox inhibitor, 50 µM) (n = 7). (**B**) Representative trace and averaged I-V plot highlighting the inability of Ang II (100 nM) to inhibit the T-type current in cells incubated with apocynin (n = 8). (**C**) Representative trace, and averaged I-V plots of the whole cell T-type current, prior to and following the application of ML171 (selective Nox 1 inhibitor, 1 µM) (n = 7). (**D**) Representative trace and average I-V plot highlighting the inability of Ang II (100 nM) to inhibit the T-type current in cells incubated with ML171 (n = 7). (**E**) Peak T-type current (pA/pF) at 5 min time intervals under control condition (n = 8) or in cells treated with either apocynin or ML171 ± Ang II (n = 7–8). *Denotes significant difference compared to control (*P < 0.05, paired t test).

AT_1_ receptors are coupled to the classic Gq pathway which mobilizes phospholipase C, fostering diacylglycerol production and the activation of PKC^17^. Figure 2 demonstrates the conventional PKC blocker Go 6976 (100 nM) had no measurable effect on basal Ba^2+^ current or the ability of Ang II to suppress Ca_V_3.x channels (from −1.524 ± 0.0559 pA/pF to 1.0914 ± 0.0442 pA/pF). Similar results were obtained using the broad-spectrum PKC inhibitor GF 109203 × (100 nM) (from −1.46 ± 0.06 pA/pF to −0.98 ± 0.033 pA/pF) (Fig. 2). Neither blockers were associated with significant change in the voltage dependence of steady state activation/inactivation (Supplementary Figure I) We subsequently addressed the involvement of Nox, a ROS generating enzyme recently tied to the AT_1_ receptor in the resistance vasculature^15^. Apocynin (50 µM), a general Nox inhibitor, had little impact on basal currents or kinetics (Fig. 3). It did, however, abolish Ang II suppression (−1.54 ± 0.08 pA/pF compared to −1.58 ± 0.061 pA/pF), highlighting a role for ROS generation. While vascular smooth muscle cells express several Nox isoforms, subtype 1 has been previously implicated in mediating Ang II effects^23,24^. Consistent with this perspective, selective inhibition of Nox 1 (ML171, 1 µM) eliminated Ang II suppression of the inward T-type current (−1.7 ± 0.07 pA/pF versus −1.75 ± 0.066 pA/pF) (Fig. 3). Similar to apocynin, ML171 had little effect on the basal current or the voltage dependence of steady state activation/inactivation (Supplementary Figure II).

**Figure 4:**
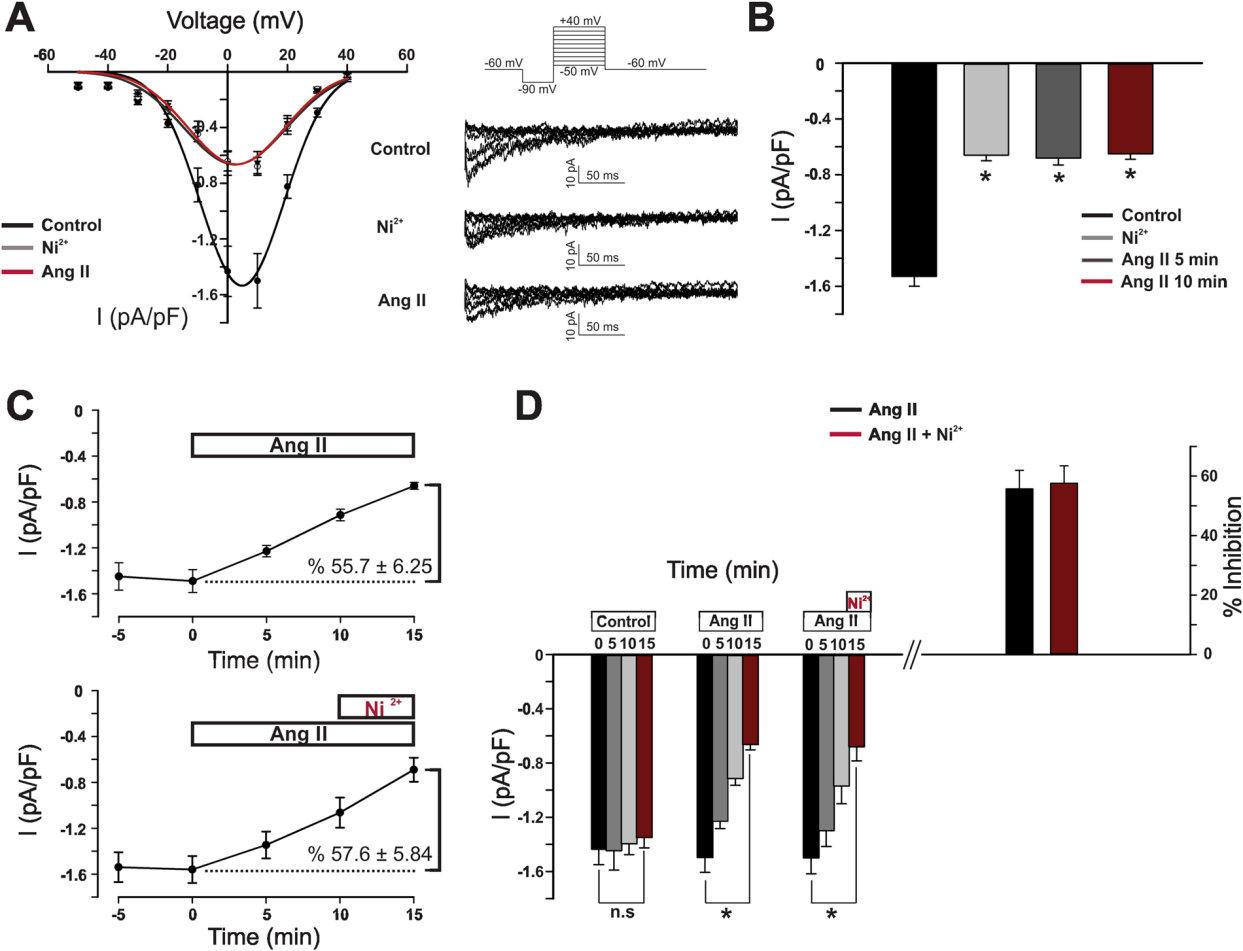
Angiotensin II selectively inhibits Ca_V_3.2 channel in cerebral myocytes. (**A**) Representative traces and average I-V plots showing the effect of Ang II (100 nM) on the residual Ni^2+^-insensitive T-type current (n = 8). (**B**) Summary data highlighting the inability of Ang II to inhibit the peak T-type current (pA/pF) in cells treated with Ni^2+^ (Ca_V_3.2 blocker, 50 µM) (n = 8). (**C**) T-type current (I_max_, pA/pF) suppression by Ang II in the absence (top, n = 8) and presence (bottom, n = 6) of Ni^2+^. (**D**) Left bar graph illustrate change in peak T-type current (5 min time intervals) under control conditions (n = 8) or in presence of Ang II ± Ni^2+^ (n = 6–8); right bar graph displays % inhibition of the T-type current. *Denotes significant difference compared to control (*P < 0.05, paired t test).

**Figure 5:**
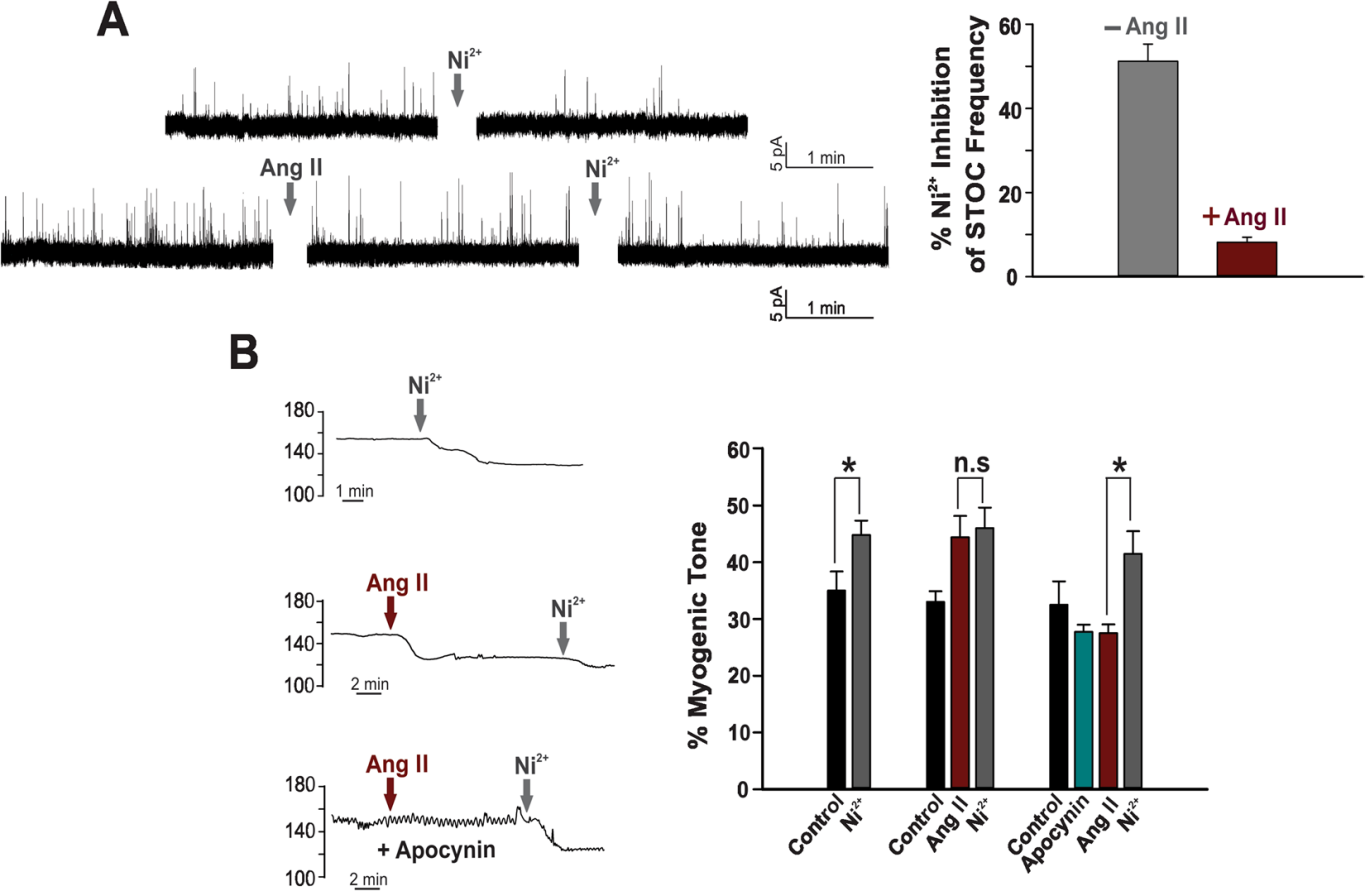
Angiotensin II abolishes Ni^2+^’s ability to inhibit STOCs and augment myogenic tone. (**A**) Representative traces and summary data showing Ni^2+^ (50 µM)-induced inhibition of STOC frequency in absence (n = 6) and presence (n = 6) of Ang II (100 nM). Perforated patch clamp electrophysiology was used to measure STOCs in cerebral arterial smooth muscle cells held at −40 mV. (**B**) Representative trace and summary data comparing the effect of Ni^2+^ on myogenic tone (60 mmHg) in absence (n = 5) and presence (n = 5) of Ang II. Ni^2+^ failed to alter arterial tone in vessels pretreated with Ang II, a phenomenon reversed with Nox inhibition with apocynin (50 µM) (n = 5). *Denotes significant difference (*P < 0.05, paired t test).

**Figure 6:**
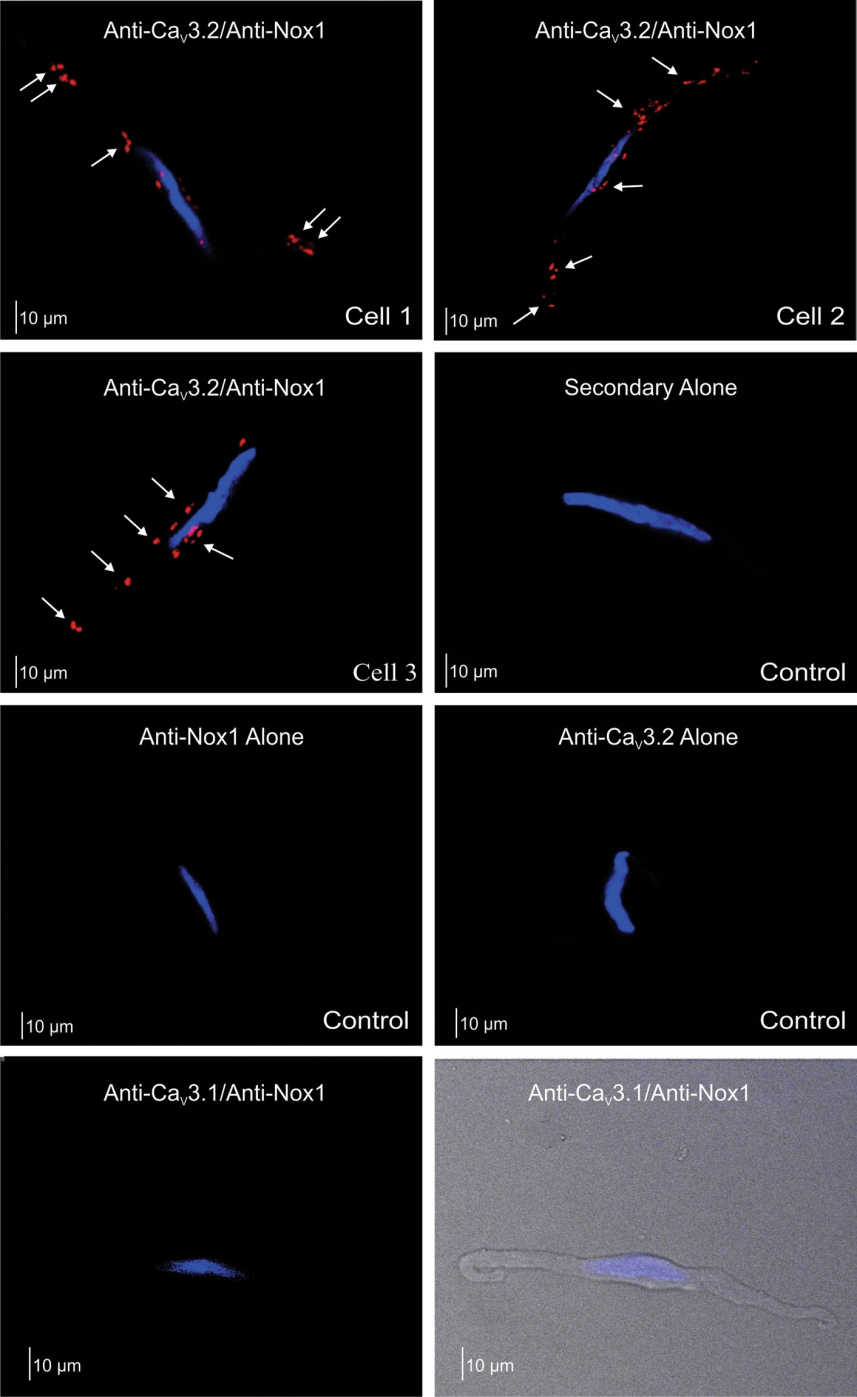
Proximity ligation assay of Ca_V_3.2 and Nox1 in cerebral arterial smooth muscle cells. Representative images revealing the close proximity (<40 nM) of Ca_V_3.2 with Nox1 as demonstrated by the presence of red fluorescent products; nuclei were labelled with DAPI (blue). A similar localization pattern between Nox1 and other vascular T-type channels (Ca_V_3.1) could not be detected. Assay controls were performed with one or both primary antibodies removed. Each experiment was performed on cells for 4 different animals; photomicrographs are representative 10–20 smooth muscle cells per group.

The T-type current in cerebral arterial smooth muscle reflects the composite activity of Ca_V_3.1 and Ca_V_3.2 channels^12^. These two individual currents can be differentiated from one another by applying low micromolar Ni^2+^, a divalent cation that selectively blocks Ca_V_3.2^8,25^. Figure 4 demonstrates that when Ni^2+^ is placed in the bath, Ang II is unable to suppress the remaining current, representative of Ca_V_3.1 channels. (−0.68 ± 0.05 pA/pF compared to −0.65 ± 0.04 pA/pF). Likewise, Ni^2+^ failed to augment T-current suppression in cells pretreated with Ang II compared to the effect of the vasoactive peptide alone (57.6 ± 5.84% versus 55.7 ± 6.25%, respectively). Selective targeting of Ca_V_3.2 was further confirmed by monitoring STOC production and noting the inability of Ni^2+^ to inhibit STOC frequency in isolated cells pretreated with Ang II (1.1 ± 0.12 Hz to 0.975 ± 0.19 Hz compared to 1.29 ± 0.095 Hz to 0.53 ± 0.092 Hz under control conditions) (Fig. 5). Functional data aligned with electrophysiological observations in that Ni^2+^ application failed to increase myogenic tone (4% change) in arteries pretreated with Ang II compared to the 25% rise under standard conditions. Intriguingly, the Ni^2+^ mediated increase in myogenic tone was restored in arteries that were pretreated with apocynin, a finding consistent with ROS modulating Ca_V_3.2 (Fig. 5). Similar results were obtained with the Nox1 inhibitor ML171 (Supplementary Figure III). Pretreating arteries with the Ni^2+^ did not alter their responsiveness to Ang II (Supplementary Figure IV). Such observations structurally imply that the ROS generating enzyme Nox 1 colocalizes with Ca_V_3.2. This was confirmed with the proximity ligation assay (Fig. 6); punctate red fluorescent product was observed in cells treated with both primary antibodies (anti Ca_V_3.2 and anti Nox1) in keeping with proteins residing within 40 nm of one another. Fluorescent product was absent in control experiments where one or both primary antibodies were omitted. Further control experiments reveal no similar co-localization pattern between Nox1 and Ca_V_3.1, a secondary T-type Ca^2+^ channel expressed in cerebral arteries (Fig. 6).

## Discussion

**Figure 7:**
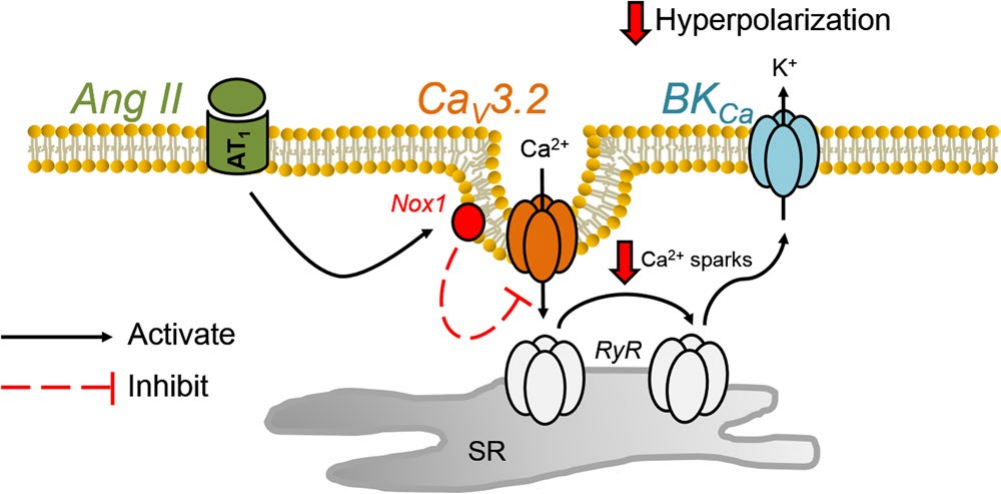
Schematic diagram highlighting the mechanism of Ang II suppression of Ca_V_3.2 channels and its functional impact on the BK_Ca_ mediated feedback in cerebral arteries.

This study examined whether and by what mechanism Ang II modulates T-type Ca^2+^ channels in cerebral arterial smooth muscle cells. This constrictor peptide was initially observed to suppress T-type channels via activation of AT_1_ receptors and a signaling pathway independent of PKC. The inhibition of Nox, a unique signaling protein involved in local ROS generation, abolished Ang II suppression. Further experiments revealed that Ang II specifically targets Ca_V_3.2 channels via Nox 1, two proteins that localize within 40 nm of one another. Functionally, Ang II mediated T-channel suppression will impair STOC production at physiological voltages, reducing negative feedback and facilitating arterial constriction. This study is the first in vascular tissue to consider T-type Ca^2+^ channels as a regulatory target of Ang II and downstream Nox signaling in the genesis of arterial constriction (Fig. 7).

### Background

Cerebral arterial tone is actively controlled by vasoactive stimuli present in the systemic circulation or locally produced within tissue^1,2^. Dynamic tuning is intimately tied to membrane potential and the gating of L-type Ca^2+^ channels which sets cytosolic [Ca^2+^] and the activity of myosin light chain kinase^26,27^. In addition to L-type channels, two T-type channels (Ca_V_3.1 and Ca_V_3.2) are expressed in cerebral arterial smooth muscle, each of which subtly contributes to tone development in a mechanistically unique manner^6,28^. Of particular note is Ca_V_3.2, a channel whose opening paradoxically limits arterial constriction by discretely triggering Ca^2+^ release from ryanodine receptors (RyR) localized to the sarcoplasmic reticulum^8^. These transient events activate BK_Ca_ channels, driving STOC production, hyperpolarizing events which attenuate arterial constriction^29^. In considering the close and evolving relationship between Ca_V_3.2 and STOC production, mechanistic questions logically arise as to the dynamic regulation of this feedback cascade. Vasoconstrictors for example, including those that circulate systemically or produced locally in the arterial wall (Ang II) suppress this cascade and facilitate arterial tone generation^19,30^. This suppression is often attributed to a direct BK_Ca_ suppression with little consideration that the upstream initiators of Ca^2+^ sparks may themselves be the target of inhibition^11,31^. It is this issue that will be addressed in the current manuscript.

### Initial Experimentation

Work began by examining the effect of Ang II on Ca_V_3.x channels expressed in cerebral arterial smooth muscle cells^6^. Patch clamp electrophysiology revealed that this vasoactive peptide suppressed the T-type current in a time dependent manner, and initiated a rightward shift trend in the voltage dependency of activation (Fig. 1). The contractile effects of Ang II are typically mediated through AT_1_ and AT_2_ receptors, with the former expressed in smooth muscle and tied to constriction, and the latter present in endothelium and intimately tied to nitric oxide production^32,33^. Consistent with the involvement of AT_1_ receptors, Losartan, a known anti-hypertensive agent, abolished Ang II suppression of the T-type current. As AT_1_ receptors are G_q_ coupled, it is logical to deduce that Ang II suppression is linked to phospholipase C-β and the mobilization of PKC, a phosphoprotein known to target multiple ion channels^17,34,35^. Surprisingly, both conventional and broad spectrum PKC inhibitor (Go 6976 and GF 109203X, respectively), previously shown to interfere with phorbol ester induced constriction, failed to abolish T-type current suppression (Fig. 2)^36^. These observations suggest that AT_1_ receptor signaling is diverse in cerebral arterial smooth muscle and that other downstream effectors are uniquely involved in targeting the T-type current.

### Involvement of Reactive Oxygen Species

Ang II application in the cerebral circulation elevates ROS production through the stimulation of the AT_1_ receptor^15,37^. While several ROS generating mechanisms are present in this vascular bed, Nox is of particular importance due to its presumed coupling with the AT_1_ receptor^15,38^ and its ability to regulate Ca^2+^ conductances including L-type Ca^2+^ channels^39^. Consistent with ROS generation impacting a broader range of Ca^2+^ permeable pores, pretreating cells with the pan selective Nox inhibitor apocynin abolished the suppressive effects of Ang II on T-type channels (Fig. 3). While impactful on Ang II mediated effects, apocynin had no measurable impact on peak inward current or activation/inactivation kinetics under basal conditions. Of the seven known Nox isoforms, three have been identified at the molecular level in cerebral arteries, two of which (Nox1 and Nox4) are expressed in vascular smooth muscle^40,41^. Observations from knockout and transgenic mice suggest that Nox1 is a key downstream target of the AT_1_ receptor and important to mediating ROS generation in vasculature^23,42,43^. In alignment with this observation, the Nox1 selective inhibitor, ML171 attenuated the ability of Ang II to suppress the T-type current in cerebral arterial smooth muscle (Fig. 3).

The T type current in vascular smooth muscle is representative of both Ca_V_3.1 and Ca_V_3.2 and in theory Ang II could be targeting one or both of the channel subtypes^6^. The two Ca_V_3.x subtypes can be differentiated from one another using Ni^2+^, a divalent cation when applied at low micromolar concentrations will selectively blocks Ca_V_3.2^12,25^. In the presence of 50 µM Ni^2+^ the addition of Ang II failed to modulate the remaining T-type current, representative of Ca_V_3.1 (Fig. 4). Ni^2+^ had no inhibitory effect in smooth muscle cells pretreated with Ang II and together both findings suggest that AT_1_ receptors and the downstream generation of ROS are selectively targeting Ca_V_3.2 channels. While the precise mechanism of inhibition remains unclear, past work in neuronal tissue has noted that oxidation of histidine residue H191 potently inhibits Ca_V_3.2 channels^44^. The selective targeting of Ca_V_3.2 has potentially important implications in regard to resident feedback loops present in cerebral arterial smooth muscle cells. In detail, Ca_V_3.2 has been reported to trigger the cytosolic gate of RyR, initiating Ca^2+^ sparks that sequentially activate BK_Ca_ channels, generating a hyperpolarization that moderates arterial constriction^8,21^. It follows that if Ang II is indeed selectively targeting Ca_V_3.2 that the channel’s ability to generate STOCs should be moderated and subject to negative feedback in presence of the peptide. Consistent with this perspective, Ni^2+^ failed to reduce STOC frequency in cells, or enhance arterial tone in vessels, superfused with Ang II (Fig. 5). Intriguingly, Ni^2+^ mediated increases in arterial tone could be restored if vessels were pretreated with apocynin or ML171, prior to Ang II addition. This particular finding further confirms a role for ROS and to our knowledge the first to demonstrate selective modulation of Ca_V_3.2 in the cerebral vasculature.

As ROS molecules are remarkably labile, targeted redox signaling can only occur if a defined enzyme generating system is compartmentalized in close apposition to the selected protein^45^. We consequently presumed, given the preceding observations, that Nox1 and Ca_V_3.2 colocalize with one another, perhaps in caveolae where both proteins have been reported to reside^8,46^. One means of detecting close interaction is to employ the proximity ligation assay, a technique where two secondary antibodies with attached DNA strands bound to two distinct primary antibodies form a circular template for DNA amplification if the targeted proteins are within 40 nM of one another^8^. Consistent with close apposition among Ca_V_3.2 and Nox1, punctate red florescence label was detected on the smooth muscle cell membrane (Fig. 6). Fluorescence labeling was absent in experiments where one or both primary antibodies were removed from the biochemical process. This localization pattern was unique to Ca_V_3.2 and didn’t extend to Ca_V_3.1 (Fig. 6), a result which highlights regulatory distinctiveness among T-type Ca^2+^ channels.

### Broader Implications

Tone development is intimately tied to changes in arterial membrane potential and the subsequent rise of intracellular [Ca^2+^] via L-type Ca^2+^ channels^26^. Membrane potential is in turn driven by a range of conductances, several of which are assigned the role of feeding back negatively upon depolarization, to prevent vessels from overly constricting^10,11,47^. As noted above, BK_Ca_ is a key feedback element and a channel whose activity is regulated indirectly by Ca_V_3.2 and a discrete influx of Ca^2+^ that triggers the cytosolic gate of RyR^8^. It is clear that Ang II suppresses Ca_V_3.2 activity and with it the resultant feedback loop, an observation with implications to vascular disease. High renin models of hypertension are typically associated with both an increase in arterial tone and a rise in ROS production^48–50^. It is intriguing to consider whether ROS generation partially drives the rise in tone through an axis sequentially entailing the AT_1_ receptor, Nox1, Ca_V_3.2, Ca^2+^ sparks and the transiently opening BK_Ca_. In considering this potential mechanism, it’s important to acknowledge that Ca_V_3.2 is not the sole conductance that triggers the opening of RyR and the generation of Ca^2+^ sparks^21^. There is a complementary role for L-type Ca^2+^ channels and the Na^+^/Ca^2+^ exchanger particularly at depolarized potentials whose resident activity may also be impacted by the hypertensive state^21^.

### Summary

Findings in this study support the emerging view that T-type Ca^2+^ channels are a rich regulatory target for vasoactive stimuli. In addition to being modulated by dilatory protein kinases, the current study shows that particular Ca_V_3.x channels are targeted by vasoconstrictors and their associated receptors/signaling pathway. This includes Nox mediated generation of ROS via the AT_1_ receptor, a process that selectively suppresses Ca_V_3.2 and along with it BK_Ca_ mediated feedback in the cerebral vasculature (Fig. 7). These findings elucidate a new mechanism of arterial tone regulation, one that could contribute to the excessive constricted state observed with advancing vascular disease.

## Materials and Methods

### Animal procedures

Animal procedures and methods were approved by the Animal Care and Use Committees at the University of Calgary and the University of Western Ontario and in accordance with the Canadian Institute of Health Research (CIHR) guidelines. Female Sprague–Dawley rats (10–12 weeks of age) were euthanized by CO_2_ asphyxiation. The brain was carefully removed and placed in cold phosphate-buffered saline (PBS, pH 7.4) solution containing (in mM): 138 NaCl, 3 KCl, 10 Na_2_HPO_4_, 2 NaH_2_PO_4_, 5 glucose, 0.1 CaCl_2_ and 0.1 MgSO_4_. Middle and posterior cerebral arteries were isolated and cut into 2–3 mm segments.

### Isolation of Cerebral Arterial Smooth Muscle Cells

Middle and posterior cerebral arteries were enzymatically digested to liberate smooth muscle cells^10^. Briefly, arterial segments were placed in an isolation medium (37 °C, 10 minutes) containing (in mM): 60 NaCl, 80 Na-glutamate, 5 KCl, 2 MgCl_2_, 10 glucose and 10 HEPES with 1 mg/ml bovine serum albumin (pH 7.4). Vessels were then exposed to a two-step digestion process that involved: 1) 14 minutes incubation in isolation medium (37 °C) containing 0.5 mg/ml papain and 1.5 mg/ml dithioerythritol; and 2) 10 minutes incubation in isolation medium containing 100 μM Ca^2+^, 0.7 mg/ml type F collagenase and 0.4 mg/ml type H collagenase. Following incubation, tissues were washed repeatedly with ice-cold isolation medium and triturated with a fire-polished pipette. Liberated cells were stored in ice-cold isolation medium for use the same day within ~5 hours.

### Electrophysiological Recordings

Whole cell currents were measured in isolated cerebral arterial smooth muscle cells. Current was recorded using an Axopatch 200B patch-clamp amplifier (Molecular Devices, Sunnyvale, CA), filtered at 1 kHz, digitized at 5 kHz, and were stored on a computer for offline analysis with Clampfit 10.3 software (Molecular Devices, Sunnyvale, CA). Whole-cell capacitance averaged 12–18 pF and was measured with the cancellation circuity in the voltage clamp amplifier. Access resistance was monitored every 60 s and cells with changes greater than 2 MΩ were excluded. To minimize offset potential (<2 mV), a 1 M NaCl–agar salt bridge between the reference electrode and the bath solution was used. All experiments were performed at room temperature (~22 °C).

For measuring Ca_V_3.x current, conventional patch-clamp electrophysiology was utilized. Briefly, recording electrodes (5–8 MΩ) were pulled from borosilicate glass microcapillary tubes (Sutter Instruments, Novato, CA) using a micropipette puller (Narishige PP-830, Tokyo, Japan), and backfilled with pipette solution containing (in mM): 135 CsCl, 5 Mg-ATP, 10 HEPES, and 10 EGTA (pH 7.2). Cells were placed in a bath solution consisting of (in mM): 110 NaCl, 1 CsCl, 10 BaCl_2_, 1.2 MgCl_2_, 10 glucose, and 10 HEPES (pH 7.4). For whole cell current recordings, smooth muscle cells were voltage clamped at a holding potential of −60 mV and subjected to a −90 mV followed by a 10 voltage step (300 ms) starting from −50 to 40 mV. I-V relationships were plotted as current density (pA/pF) at the different voltage steps. Voltage dependence of steady-state inactivation was assessed by a step protocol: (a) prepulse to −90 mV (300 ms); (b) stepping from −70 to 20 mV (10 mV interval, 1.5 s each); (c) hyperpolarizing back to −90 mV (10 ms); and (d) stepping to a test voltage 10 mV (200 ms). Whole cell current elicited by the test voltage was normalized to maximal current to plot % I/I_max_ versus different voltage steps. The voltage dependence of activation was evaluated by monitoring isochronal tail currents. More specifically, cells clamped at −60 mV were subjected to a prepulse (−90 mV; 300 ms) followed by voltage steps ranging from −80 to 40 mV (10 mV interval, 50 ms each), and then a final hyperpolarizing test pulse (−90 mV; 200 ms) to evoke tail currents. Normalized tail currents (% I/I_max_) were plotted versus the voltage steps. All recordings were performed in the presence of 200 nM nifedipine, a concentration previously shown to fully block Ca_V_1.2 channels without affecting Ca_V_3.x isoforms^12^.

For STOC recordings, perforated patch-clamp electrophysiology was used. The bath solution consisted of (in mM): 134 NaCl, 4 KCl, 2 MgCl_2_, 2 CaCl_2_, 10 glucose, and 10 HEPES (pH 7.4). The pipette solution contained (in mM): 110 K aspartate, 30 KCl, 10 NaCl, 2 MgCl_2_, 10 HEPES, and 0.05 EGTA (pH 7.2) with 250 µg/ml amphotericin B. BK_Ca_ currents (STOCs) were recorded while the cells were held at a membrane potential of −40 mV. Threshold for STOC detection was ~3 times BK_Ca_ single channel conductance.

### Vessel Myography

Arterial segments were mounted in a customized arteriograph and superfused with warm (37 °C) physiological salt solution (PSS; pH 7.4; 21% O_2_, 5% CO_2_, balance N_2_) containing (in mM): 119 NaCl, 4.7 KCl, 20 NaHCO_3_, 1.1 KH_2_PO_4_, 1.2 MgSO_4_, 1.6 CaCl_2_ and 10 glucose. To limit the influence of the endothelium, air bubbles were passed through the vessel lumen (1 min); successful removal was confirmed by the loss of bradykinin-induced dilation. Arteries were equilibrated at 15 mmHg and contractile responsiveness assessed by brief application of 60 mM KCl. Following equilibration, intravascular pressure was elevated from 20 to 60 mmHg, and arterial external diameter was monitored under control conditions and in the presence of Ang II and/or Ni^2+^ (Ca_V_3.2 blocker). Maximal arterial diameter was subsequently assessed in Ca^2+^-free PSS (zero externally added Ca^2+^ plus 2 mM EGTA). An additional set of experiments was conducted on vessels preincubated with apocynin (NADPH oxidase inhibitor) using the same experimental protocol. Percent myogenic tone was calculated as follows: % myogenic tone = 100 * (D_0_ − D)/D_0_; where D is external diameter under control conditions (Ca^2+^ PSS) or treated conditions, and D_0_ is external diameter in Ca^2+^-free PSS.

### Proximity Ligation Assay (PLA)

The Duolink *in situ* PLA detection kit was employed using freshly isolated smooth muscle cells. Briefly, cells were first fixed in PBS containing 4% paraformaldehyde (15 min), and then incubated with PBS containing 0.2% tween (15 min) for permeabilization. Cells were washed with PBS, blocked by Duolink blocking solution (1 hr), and incubated overnight with primary antibodies (mouse anti-Ca_V_3.2 or mouse anti Ca_V_3.1 and rabbit anti Nox1) in Duolink antibody diluent solution at 4 °C. Control experiments employed no primary antibody or one primary antibody. Cells were then washed with Duolink washing solution, and labelled with Duolink PLA PLUS and MINUS probes for 1 hr (37 °C). The secondary antibodies of PLA PLUS and MINUS probes are attached to synthetic oligonucleotides that hybridize if present in close proximity (<40 nm). The hybridized oligonucleotides are then ligated and subjected to amplification. The amplified products extending from the oligonucleotide arm of the PLA probe were detected using far red fluorescent fluorophore-tagged, complementary oligonucleotide sequences and Leica TCS SP8 confocal microscope.

### Chemicals and Statistical Analysis

GF 109203X was purchased from Calbiochem. Ang II, losartan, Go 6976, apocynin, and ML-171 were obtained from Tocris. Primary antibodies against Ca_V_3.2, Ca_V_3.1 and Nox1 were purchased from Novus Biologicals. Duolink PLA detection kit and all other chemicals were obtained from Sigma Aldrich. Data are expressed as means ± S.E., and n indicates the number of cells or arteries or animals. Paired t-test was performed to compare the effects of a given condition/treatment on whole cell current or arterial diameter. P values ≤ 0.05 were considered statistically significant.

### Data Availability

The datasets generated during and/or analyzed during the current study are available from the corresponding author in a reasonable request.

## Electronic supplementary material

Supplementary Material
