# Supplementary material for: Reactive Oxygen Species Mediate the Suppression of Arterial Smooth Muscle T-type Ca2+ Channels by Angiotensin II
Source: Sci Rep. 2018 Feb 22;8:3445. doi: 10.1038/s41598-018-21899-5 (PMC5823855; doi:10.1038/s41598-018-21899-5)
Supplement: Supplementary file 1 — Supplementary Material [file 41598_2018_21899_MOESM1_ESM.pdf]

Reactive Oxygen Species Mediate the Suppression of Arterial Smooth Muscle T-type Ca^{2+} Channels by Angiotensin II

Ahmed M. Hashad [1], Maria Sancho [2], Suzanne E. Brett[2], Donald G. Welsh [1, 2]

[1]Dept. of Physiology & Pharmacology, Hotchkiss Brain and Libin Cardiovascular Institutes, University of Calgary, Alberta, Canada.

[2]Dept. Physiology & Pharmacology, University of Western Ontario, London, Ontario, Canada.

Correspondence to:

Donald G. Welsh
Dept. Physiology and Pharmacology, Robarts Research Institute
Room 4245C, London, Ontario, Canada. N6A 5B7.
Tel 519-931-5777 ext. 25330
Email: dwelsh@robarts.ca.

Figure I

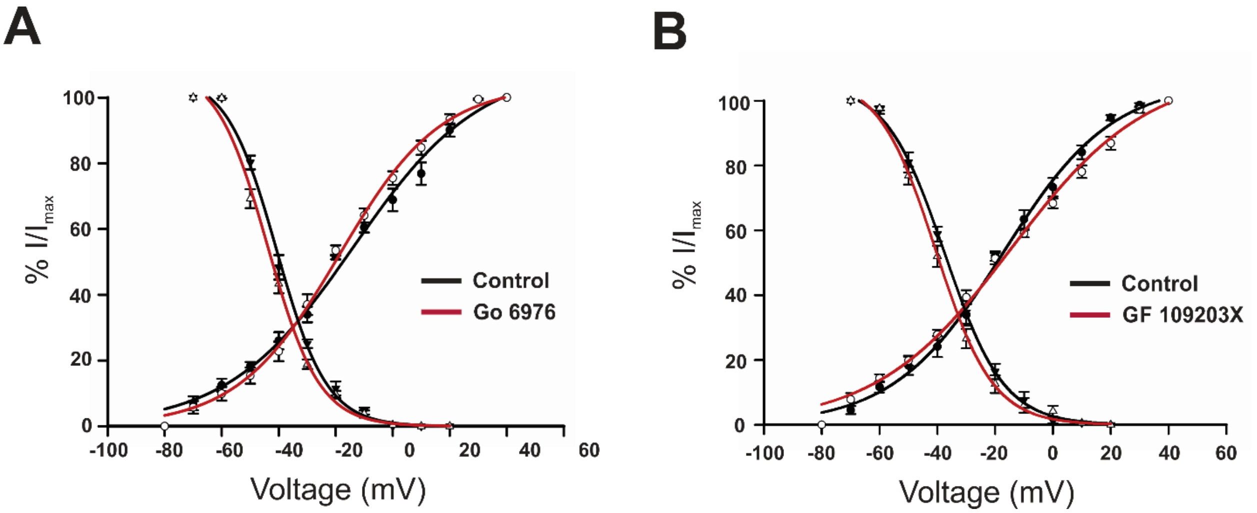

Supplementary Figure I. Protein Kinase C (PKC) inhibitors do not alter the voltage dependence of activation and steady state inactivation of T-type Ca^{2+} channels. Voltage dependence of activation and steady state inactivation of the whole cell T-type current, prior to and following the application of (A) Go 6976 (conventional PKC inhibitor, 100 nM; n=8) or (B) GF 109203X (non-selective PKC inhibitor, 100 nM; n=9).

Figure II

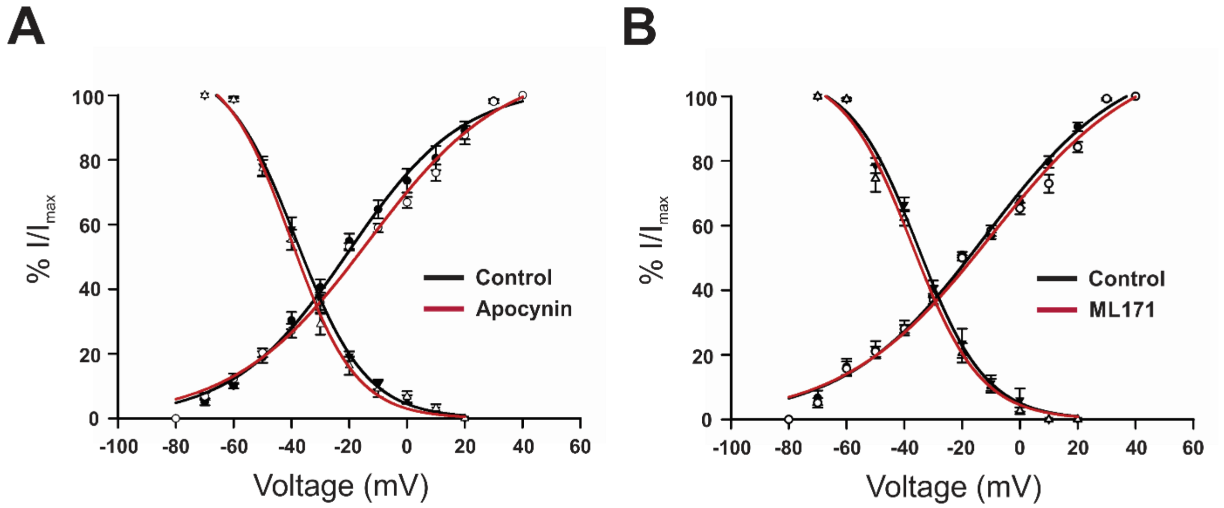

Supplementary Figure II. NADPH oxidase (Nox) inhibitors do not alter the voltage dependence of activation and steady state inactivation of T-type Ca^{2+} channels. Voltage dependence of activation and steady state inactivation of the whole cell T-type current, prior to and following the application of (A) apocynin (non-selective Nox inhibitor, 50 nM; n=7) or (B) ML171 (selective Nox 1 inhibitor, 1 µM) (n=7).

Figure III

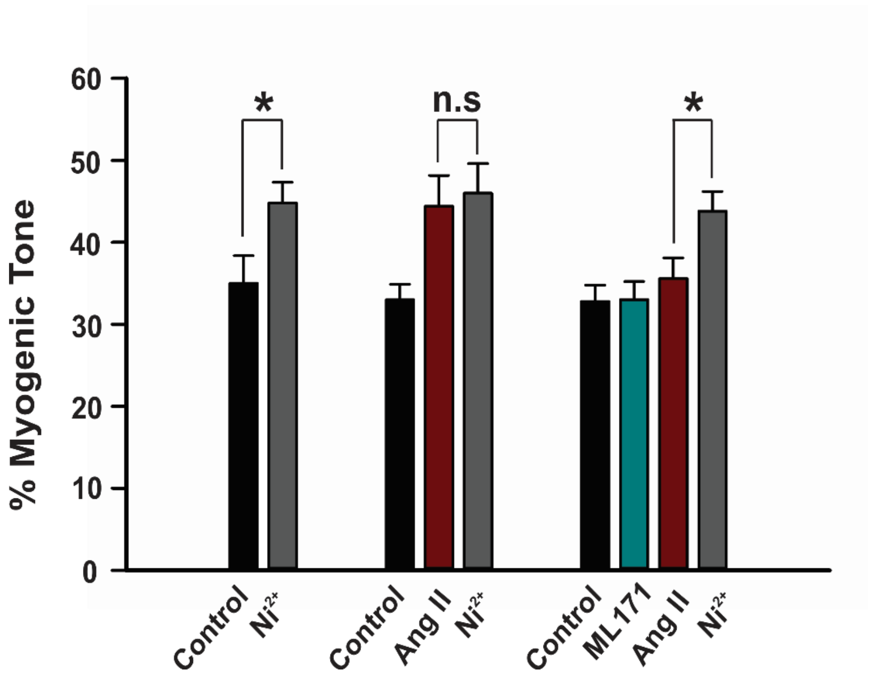

Supplementary Figure III. An NADPH oxidase (Nox) inhibitor restores the Ni^{2+} mediated augmentation of myogenic tone in vessels pretreated with Ang II. Summary data comparing the effect of Ni^{2+} on myogenic tone (60 mmHg) in absence (n=5) and presence (n=5) of the Nox1 inhibitor ML171. Ni^{2+} failed to alter arterial tone in vessels pretreated with Ang II, a phenomenon reversed with Nox1 inhibition with ML171 (1 μM) (n=5). * denotes significant difference (*$P<0.05$, paired t test).

Figure IV

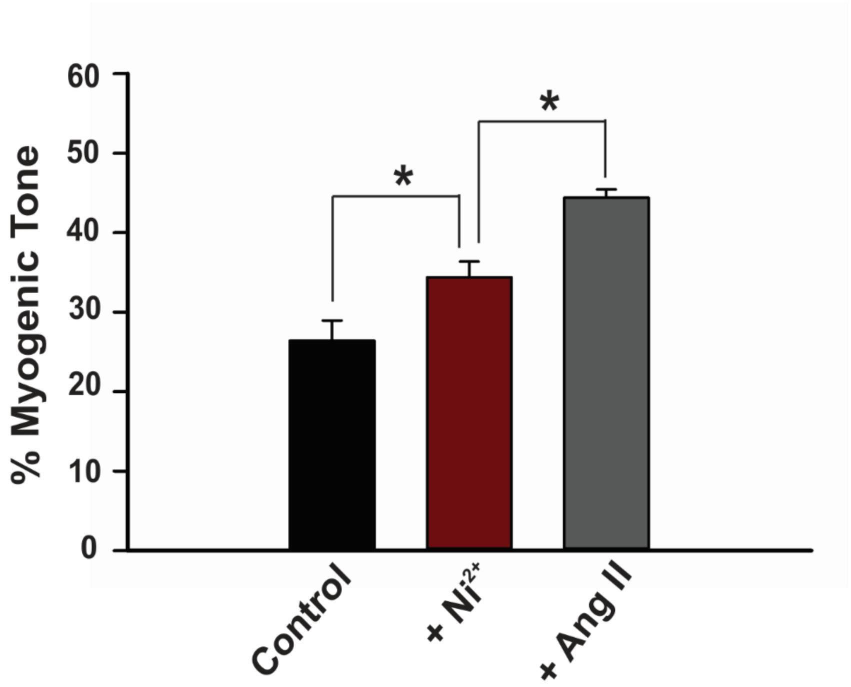

Supplementary Figure IV. Ang II constricted cerebral arteries preincubated with Ni^{2+}. Summary data highlighting the responsiveness of isolated arteries to Ang II (100 nM) under conditions in which $Ca_V3.2$ is blocked by Ni^{2+} (50 µM). Arteries (n=5) were pressurized to 60 mmHg. * denotes significant difference (*P<0.05, paired t test).
